# Performance of a photoelectron momentum microscope in direct- and momentum-space imaging with ultraviolet photon sources

**DOI:** 10.1107/S1600577523009761

**Published:** 2024-01-01

**Authors:** Tzu-Hung Chuang, Chuan-Che Hsu, Wei-Sheng Chiu, Jyun-Syong Jhuang, I-Chun Yeh, Ruei-San Chen, Shanjr Gwo, Der-Hsin Wei

**Affiliations:** a National Synchrotron Radiation Research Center, Hsinchu, Taiwan; b Graduate Institute of Applied Science and Technology, National Taiwan University of Science and Technology, Taipei, Taiwan; c Department of Physics, National Tsing-Hua University, Hsinchu, Taiwan; d Department of Physics, National Sun Yat-sen University, Kaohsiung, Taiwan; ESRF – The European Synchrotron, France

**Keywords:** momentum microscope, photoemission, electronic structure, Fermi contour, Rashba-splitting

## Abstract

The Photoelectron-Related Image and Nano-Spectroscopy (PRINS) endstation located at the Taiwan Photon Source beamline 27A2 houses a photoelectron momentum microscope capable of performing direct-space imaging, momentum-space imaging and photoemission spectroscopy with position sensitivity. Here, the performance of this microscope is demonstrated using two in-house photon sources – an Hg lamp and He(I) radiation – on a standard checkerboard-patterned specimen and an Au(111) single crystal, respectively. By analyzing the intensity profile of the edge of the Au patterns, the Rashba-splitting of the Au(111) Shockley surface state at 300 K, and the photoelectron intensity across the Fermi edge at 80 K, the spatial, momentum and energy resolution were estimated to be 50 nm, 0.0172 Å^−1^ and 26 meV, respectively. Additionally, it is shown that the band structures acquired in either constant energy contour mode or momentum-resolved photoemission spectroscopy mode were in close agreement.

## Abstract 2

A microscope hosted at Taiwan Photon Source beamline 27A2 that packs the functions of photoemission electron microscopy and angle-resolved photoemission spectroscopy into one single instrument is presented.

## 1. Introduction

Surfaces of bulk or low-dimensional materials are playing fields for scientific discovery and technological advances. However, a deep understanding of the materials’ electronic structures is required to initiate these endeavors. Because the materials under study can have various lateral dimensions and inhomogeneity, it is highly appreciated for any detection scheme to include features such as microscopy and position sensitivity.

Photoemission electron spectroscopy (PES) is a primary tool for probing the electronic structures of materials. After electrons in a solid receive a high enough energy from the photons to break free into the vacuum, PES measures the kinetic energies of emitted electrons to determine their respective binding energies. For a crystalline solid, the periodicity and symmetry of the lattice impose constraints on the electrons requiring them to occupy specific energy states and possess a permissible crystal momentum (Suga *et al.*, 2021 ▸). Since crystal momentum is a momentum-like vector, a common approach to probe a crystal’s band structure is angle-resolved photoemission spectroscopy (ARPES) which records the electron emission intensity as a function of the emission angle (Sobota *et al.*, 2021 ▸).

In this report, we demonstrate using photoelectron momentum microscopy (MM) to map the band structure of crystalline surfaces. This approach eliminates the requirement for sample rotation and prior knowledge of the surface crystal symmetry, and it has been realized by different types of MM (Kotsugi *et al.*, 2003 ▸; Krömker *et al.*, 2008 ▸; Tusche *et al.*, 2015 ▸; Schönhense *et al.*, 2020 ▸). The Photoelectron-Related Image and Nano-Spectroscopy (PRINS) endstation established at the Taiwan Photon Source (TPS) beamline 27A2 (Shiu *et al.*, 2023 ▸) is now hosting a photoelectron-based MM, which is equipped with an imaging-type electron column integrated with a single hemispherical electron energy analyzer (HEA) and an imaging spin filter. Detailed descriptions of both single- and double-hemisphere MM have been previously described (Tusche *et al.*, 2015 ▸, 2019 ▸; Suga & Tusche, 2015 ▸; Schönhense *et al.*, 2020 ▸; Matsui *et al.*, 2020 ▸, 2023 ▸). Briefly, the integration of an HEA and an imaging electron column makes it possible to acquire both photoelectron-based spectroscopy and images from the same instrument. By taking a series of direct-space and momentum-space images at selected kinetic energies, position-resolved and momentum-resolved PES can be extracted, respectively.

In the following section, we introduce the system configuration of the MM at the TPS 27A2. All photoemission experiments described in this report used either an Hg arc lamp or He(I) radiation from a commercial UVS-300 (SPECS GmbH) discharge lamp. We obtained a series of direct-space or photoemission electron microscopy (PEEM) images from a standard checkerboard patterned sample illuminated by an Hg lamp to demonstrate the imaging capabilities at different magnification settings, fields of view (FoVs) and ultimate spatial resolution in PEEM mode. Then, a series of momentum-space images were measured from an Au(111) single crystal illuminated with He(I) radiation at different kinetic energies to showcase the microscope’s capability for probing the electronic structures. The energy resolution was estimated by scanning through the Fermi edge of Au(111), while the *k*-space resolution was examined through Rashba-splitting of the Au(111) Shockley surface state. Finally, the microscope was set to ARPES mode, and a conventional ARPES spectrum was recorded to compare with the one obtained using constant energy contour mode.

## 2. System configuration

**Figure 1:**
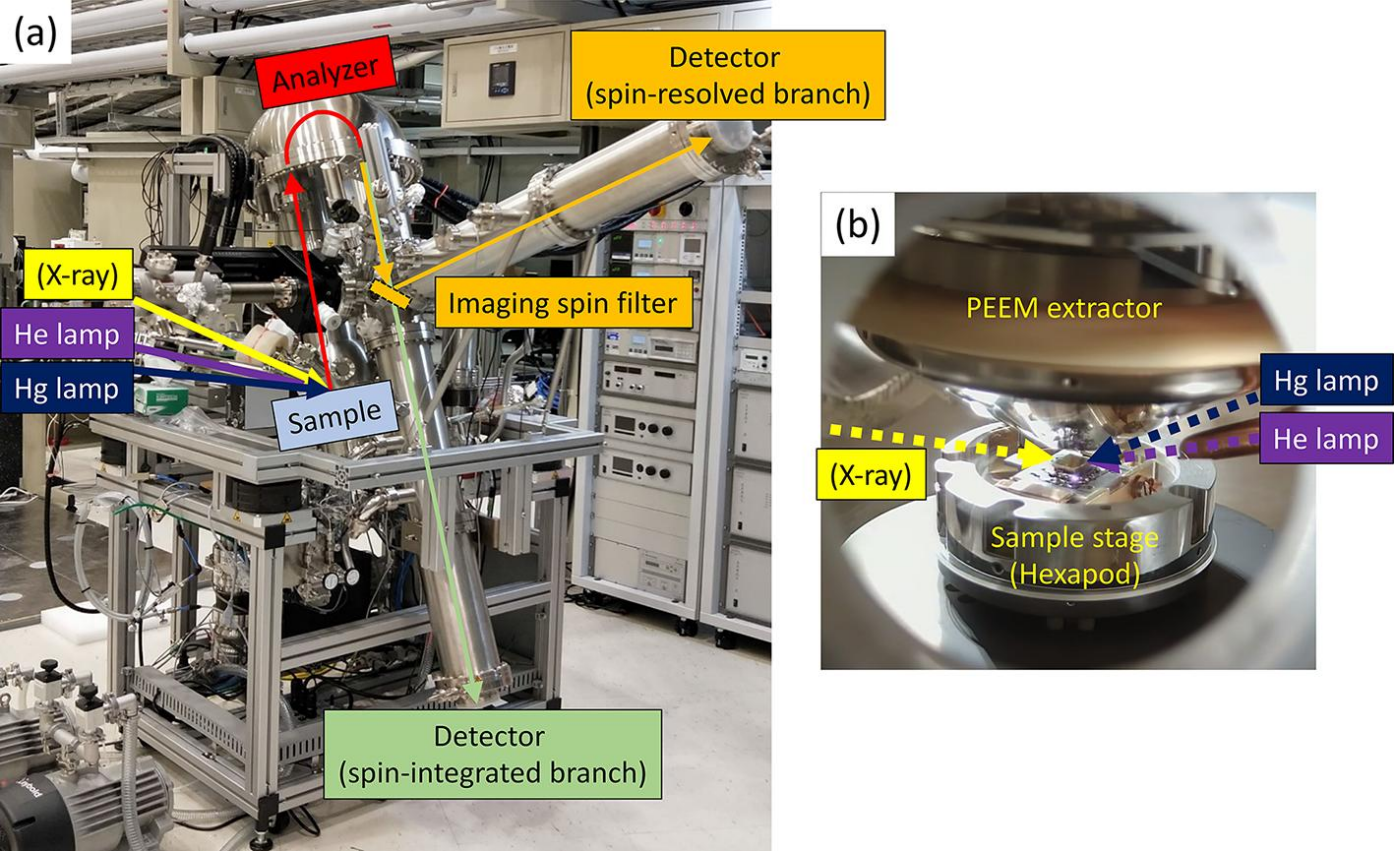
(*a*) Configuration of the MM system at the TPS 27A2. The green and orange arrows indicate the beam paths of photoelectrons in either the spin-integrated or spin-resolved branches, respectively. (*b*) Image captured from inside the measurement chamber which comprises the hexapod sample stage, the extractor lens and the capillary of the He lamp.

Offline commissioning of the MM hosted at TPS 27A2 was initiated in 2022. The KREIOS 150 MM system with single hemisphere and an imaging spin filter manufactured by SPECS GmbH has been tested using two in-house ultraviolet (UV) photon sources. Fig. 1 ▸(*a*) shows the system at the TPS 27A2 endstation after assembly, and Fig. 1 ▸(*b*) is an image taken at the sample position inside the measurement chamber, which comprises a hexapod sample stage, an extractor lens and the capillary of the He discharge lamp. In addition to the synchrotron soft X-rays, the two in-house UV photon sources include an Hg arc lamp with a photon energy of 4.9 eV and a beam size larger than 20 mm and an He discharge lamp with a photon energy of 21.2 eV for He(I) and 40.8 eV for He(II) with a beam size of about 700 µm. All photon sources were set to an incident angle of 22° relative to the sample surface and the sample surface normal aligns parallel to the optical axis of the extractor as seen in Fig. 1 ▸(*b*). The hexapod sample stage possesses six degrees of freedom: *X*, *Y*, *Z*, azimuthal rotation, *X*-axis tilting and *Y*-axis tilting. This allows proper alignment of the sample surface normal to the optical axis of the microscope. Both liquid helium (20 K) and liquid nitrogen (80 K) are available for low-temperature experiments. After excitation by photons, the photoelectrons are extracted by an extractor lens using a high voltage, typically 10–15 kV, allowing extraction of photoelectrons with emission angles up to ±90°. Thereafter, direct-space images or momentum-space images can be recorded depending on the choice of setting on the imaging optics. The HEA, with a mean radius of 150 mm, serves as an energy filter to reduce the chromatic aberrations of the microscope. The two orthogonal imaging columns connected after the HEA are used for projecting and recording either spin-resolved (orange arrows) or spin-integrated (green arrows) images. Switching between these modes is achieved by inserting (for spin-resolved) or retracting (for spin-integrated) an imaging spin filter, which is an Au/Ir(001) single crystal, along the beam path. The commissioning of the spin-resolved experiments will be demonstrated elsewhere.

## 3. Direct-space imaging

**Figure 2:**
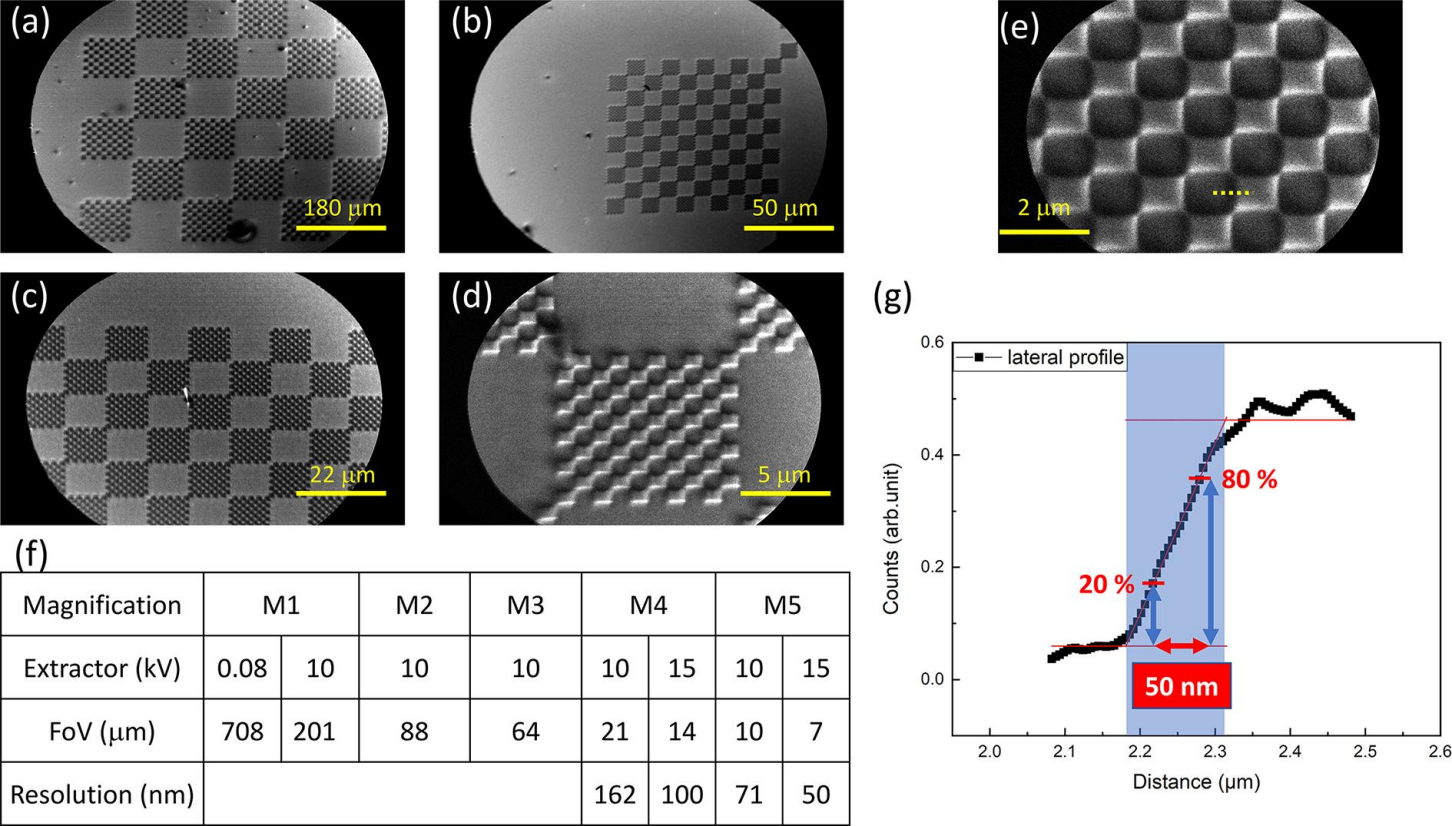
(*a*)–(*e*) Direct-space images on the checkerboard-pattered sample illuminated by an Hg lamp with various magnification settings and FoVs. (*f*) Table of magnification settings, corresponding FoVs and PEEM resolution with different extractor voltages. (*g*) Intensity profile along the edge of the Au patterns [dashed line marked in (*e*)] for estimating the PEEM resolution.

We utilized checkerboard-patterned Au thin films deposited on a Si substrate as standard samples to demonstrate the direct-space imaging capability of the MM system illuminated by an Hg lamp. Fig. 2 ▸ shows a series of energy-filtered PEEM images of these checkerboard patterns at different FoVs, ranging from around 708 µm [Fig. 2 ▸(*a*)] to 7 µm [Fig. 2 ▸(*e*)]. The contrast in these images predominantly arises from the variance in the work function between the Au films and Si substrate. The size of the FoV is typically determined by the magnification settings (M1–M5) of the imaging lens, the extractor voltage and the size of the field aperture (FA), which was set to a diameter larger than the maximum FoV during the demonstration shown in Fig. 2 ▸. Fig. 2 ▸(*f*) displays a table indicating different FoVs and the corresponding spatial resolution at different magnification settings and extractor voltages. The ultimate spatial resolution in PEEM mode (PEEM resolution) of the microscope was estimated by the intensity profile at the edge of the Au patterns and was found to be 50 nm as shown in Fig. 2 ▸(*g*).

## 4. Momentum-space imaging

**Figure 3:**
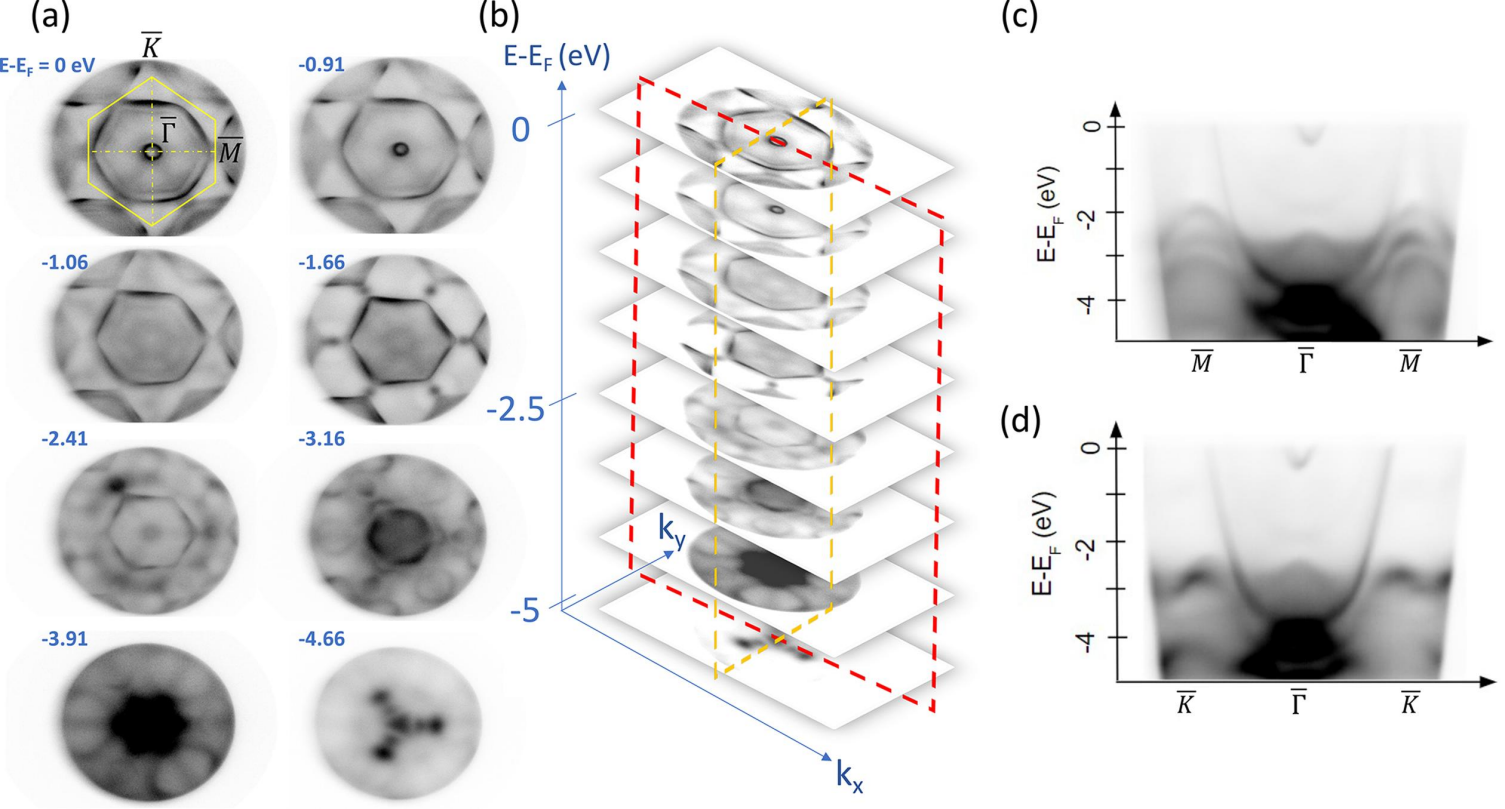
(*a*) A series of momentum images recorded at different kinetic energies obtained from an Au(111) surface at 300 K with a magnification setting of M2 and the extractor voltage of 10 kV. (*b*) Stacking of a series of momentum-space images of (*a*) to construct a 3D dataset of (*k*_*x*_, *k*_*y*_, *E*_*k*_). (*c*) Slice of the 3D dataset along the 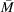 – 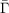 – 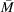 direction [red dashed line in (*b*)]. (*d*) Slice of the 3D dataset along the 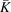 – 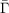 – 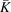 direction [orange dashed line in (*b*)].

Momentum-space imaging was examined by measuring an Au(111) surface using He(I) radiation with a photon energy of 21.2 eV. Fig. 3 ▸(*a*) shows a series of momentum-space images of Au(111) recorded using photoelectrons of different kinetic energies at 300 K. The high symmetry points of the Brillouin zone are labeled on the momentum-space image of the Fermi surface (*E* − *E*_F_ = 0 eV). It should be noted that the FoVs in momentum-space mode shown in Fig. 3 ▸(*a*) were all larger than the first Brillouin zone, where a magnification setting of M2 was applied and the extractor voltage was 10 kV. The size of the region of interest (ROI) during the momentum-space imaging of Au(111) was determined to be around 30 µm. This value was derived from the size of the FA (300 µm) divided by the lateral magnification (around 10) from the sample to the FA, therefore the ROI can be adjusted by changing these variables. The smallest ROI achieved by the single hemispherical MM was less than 1 µm (Schönhense *et al.*, 2021 ▸; Fedchenko *et al.*, 2022 ▸). By stacking a series of momentum images taken at different kinetic energies, a three-dimensional (3D) data set (*k*_*x*_, *k*_*y*_, *E*_*k*_) can be constructed as shown in Fig. 3 ▸(*b*). The kinetic energy relative to the Fermi surface was measured from −5 to 0 eV in increments of 20 meV. Thereafter, the electronic band structure along high-symmetry directions can be extracted simultaneously from this 3D data set, for example by slicing along the 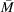 – 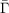 – 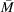 direction as shown in Fig. 3 ▸(*c*) or slicing along the 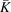 – 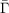 – 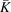 direction as shown in Fig. 3 ▸(*d*). It should be noted that we present only raw data here which have not undergone image corrections or contrast enhancement.

**Figure 4:**
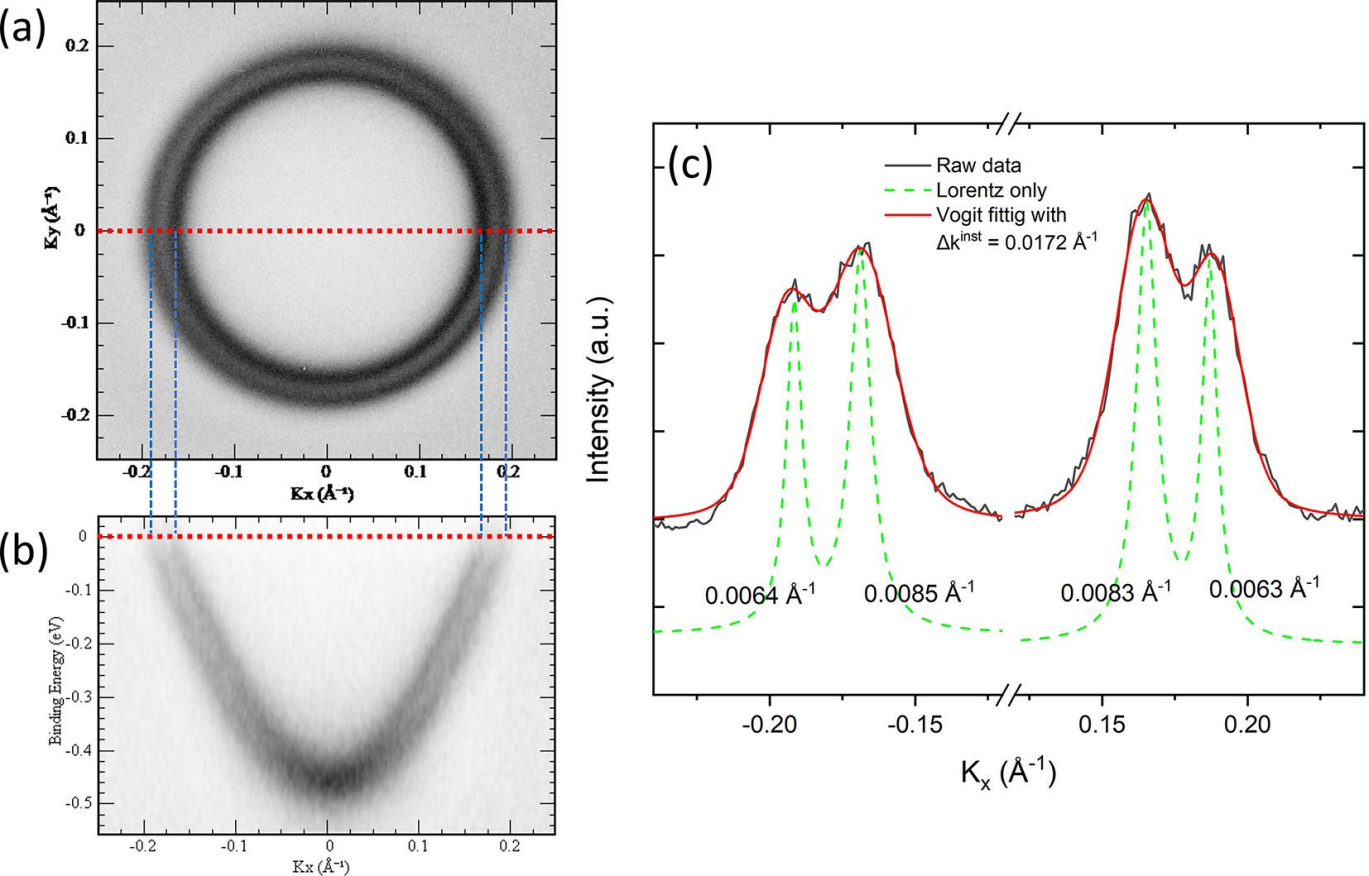
(*a*) Rashba-splitting of the Shockley surface state of Au(111) at the Fermi energy measured at 300 K with a magnification setting of M4 and an extractor voltage of 10 kV. (*b*) Energy dispersion of the surface state obtained at *K*_*y*_ = 0 slicing through the data shown in (*a*). (*c*) Intensity profile at *K*_*y*_ = 0 fitted to a Voigt function (red solid line) with 0.0172 Å^−1^ Gaussian broadening and the Lorentzian contribution (dashed line).

The momentum-space image can be examined in more detail by electron-optically selecting a larger magnification. The Shockley surface state of Au(111) is well known for its Rashba-type splitting due to strong spin–orbit coupling (LaShell *et al.*, 1996 ▸; Reinert *et al.*, 2001 ▸; Reinert, 2003 ▸; Henk *et al.*, 2003 ▸). The splitting generates two sub-bands separated in the momentum-space by *K* = 0.025 Å^−1^. To test the ultimate momentum-space resolution of the momentum microscope, the surface state of Au(111) close to the 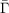 -point was magnified with a magnification setting of M4, as shown in Fig. 4 ▸(*a*). We successfully resolved the splitting of the surface state of Au(111) at room temperature, where two concentric circles at the (*K*_*x*_, *K*_*y*_) image at the Fermi surface are clearly demonstrated. Furthermore, the energy dispersion of the surface state close to the Fermi level is also shown in Fig. 4 ▸(*b*). The intensity profile of Fig. 4 ▸(*a*) at *K*_*y*_ = 0 is plotted in Fig. 4 ▸(*c*) to estimate the *k*-space resolution. The raw data (black solid line) were fitted with a Voigt function, a convolution of Gaussian broadening contributed from the instrumental resolution and a Lorentzian contribution (green dashed line) from the finite lifetime of the initial and final states in the photoemission process (Smith *et al.*, 1993 ▸; Sánchez-Royo *et al.*, 2001 ▸). According to the best fit (red solid line), our microscope’s momentum resolution at 300 K is 0.0172 Å^−1^, which is the full width at half-maximum (FWHM) used in the Gaussian function.

**Figure 5:**
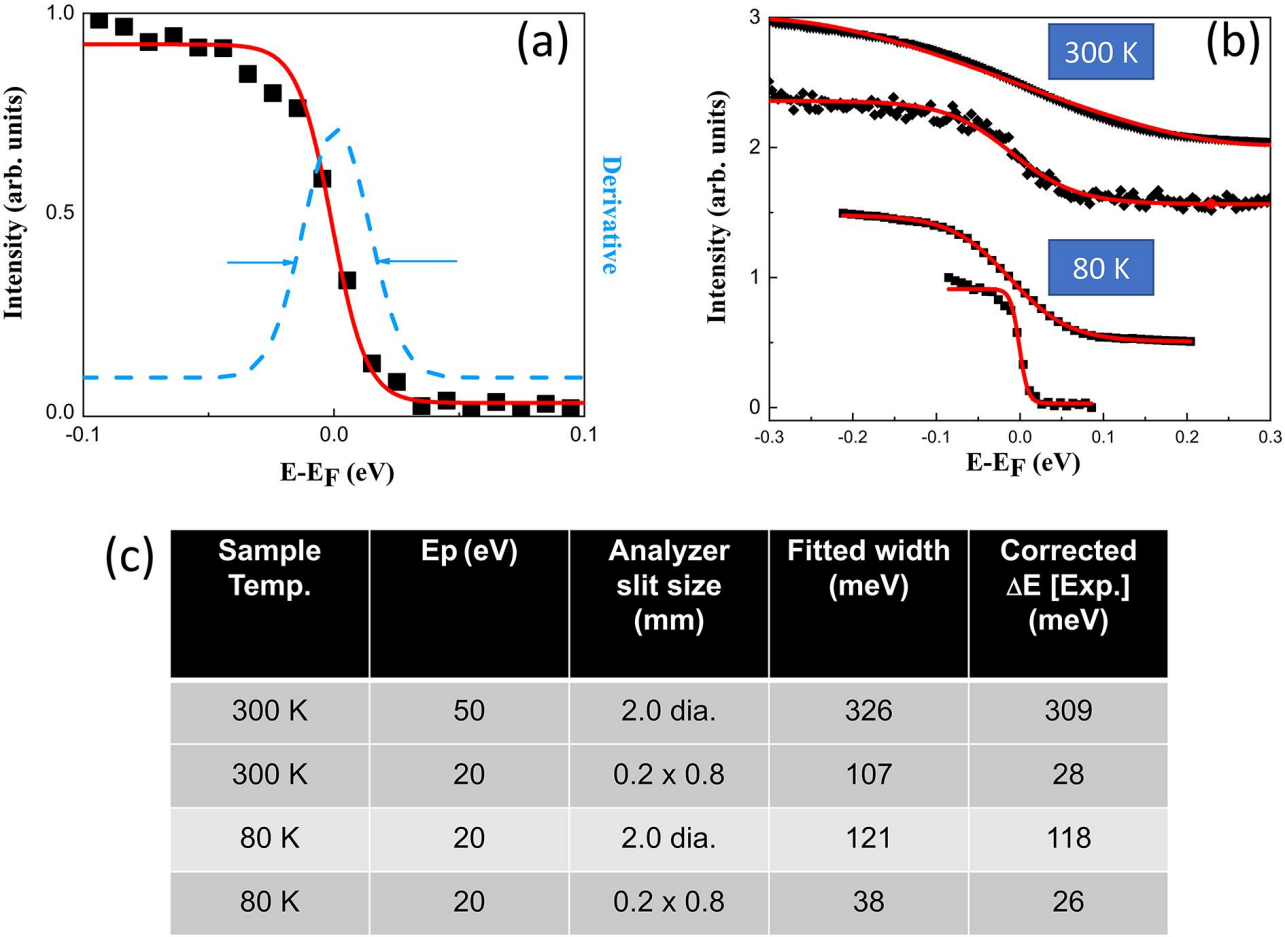
(*a*) Measured intensity profiles of the Au(111) momentum images across the Fermi energy at 80 K with the pass energy set at 20 eV and the size of the entrance slit set at 0.2 mm × 0.8 mm. The red solid line is fit to the intensity profile using the Boltzmann function, and its first derivative is shown by the blue dashed line. (*b*) Measured intensity profiles across the Fermi energy at 300 K (upper two profiles) and their fits with the analyzer pass energy and the size of entrance slit set at 50 eV and 2.0 mm in diameter or 20 eV and 0.2 mm × 0.8 mm, respectively. Intensity profiles across the Fermi energy at 80 K (lower two profiles) and their fits with the pass energy set at 20 eV and the size of entrance slit set at either 2.0 mm in diameter or 0.2 mm × 0.8 mm. (*c*) Table of the experimental energy resolution at various sample temperatures, pass energies (*E*_p_) and analyzer slit sizes.

Furthermore, the instrumental energy resolution was estimated from the intensity profiles of multiple series of momentum images recorded within an energy window across the Fermi edge at 300 K and 80 K. Fig. 5 ▸(*a*) shows the intensity profile recorded at 80 K with the pass energy and the entrance slit of HEA set at 20 eV and 0.2 mm × 0.8 mm, respectively. The profile was fitted with a Boltzmann function indicated by the red solid line. Subsequently, the fitted width (*w*_fit_) was estimated at about 38 meV by taking the FWHM of the first derivative of the fitted profile shown by the blue dashed line. Since the thermal broadening (*w*_F_ = 28 meV at 80 K) is included in *w*_fit_, the corrected instrumental resolution calculated by 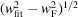 is 26 meV. Fig. 5 ▸(*b*) shows four intensity profiles across the Fermi energy measured at 300 K (upper two profiles) and 80 K (lower two profiles). The upper two profiles were recorded at 300 K with the analyzer pass energy and size of the entrance slit set at 50 eV and 2.0 mm in diameter or 20 eV and 0.2 mm × 0.8 mm, respectively. The lower two profiles were recorded at 80 K with the pass energy set to 20 eV and the size of entrance slit set at either 2.0 mm in diameter or 0.2 mm × 0.8 mm. Fig. 5 ▸(*c*) summarizes *w*_fit_ for different configurations, and the corrected experimental resolution, where the thermal broadening is de-convoluted. The best energy resolution achieved by the single-hemisphere MM with a larger radius and lower pass energy of the hemisphere was 4.2 meV (Schönhense *et al.*, 2021 ▸). In addition, for the double-hemisphere tandem MM using the same radius and similar pass energy of the hemisphere as the current report, a 13 meV energy resolution was achieved (Tusche *et al.*, 2019 ▸). Detailed information regarding the lateral, momentum and energy resolution as well as the angular filling factors, which determine the performance of the single-hemisphere MM, has been previously described in the literature (Schönhense *et al.*, 2020 ▸).

## 5. Switching between constant energy contour mode and ARPES mode

**Figure 6:**
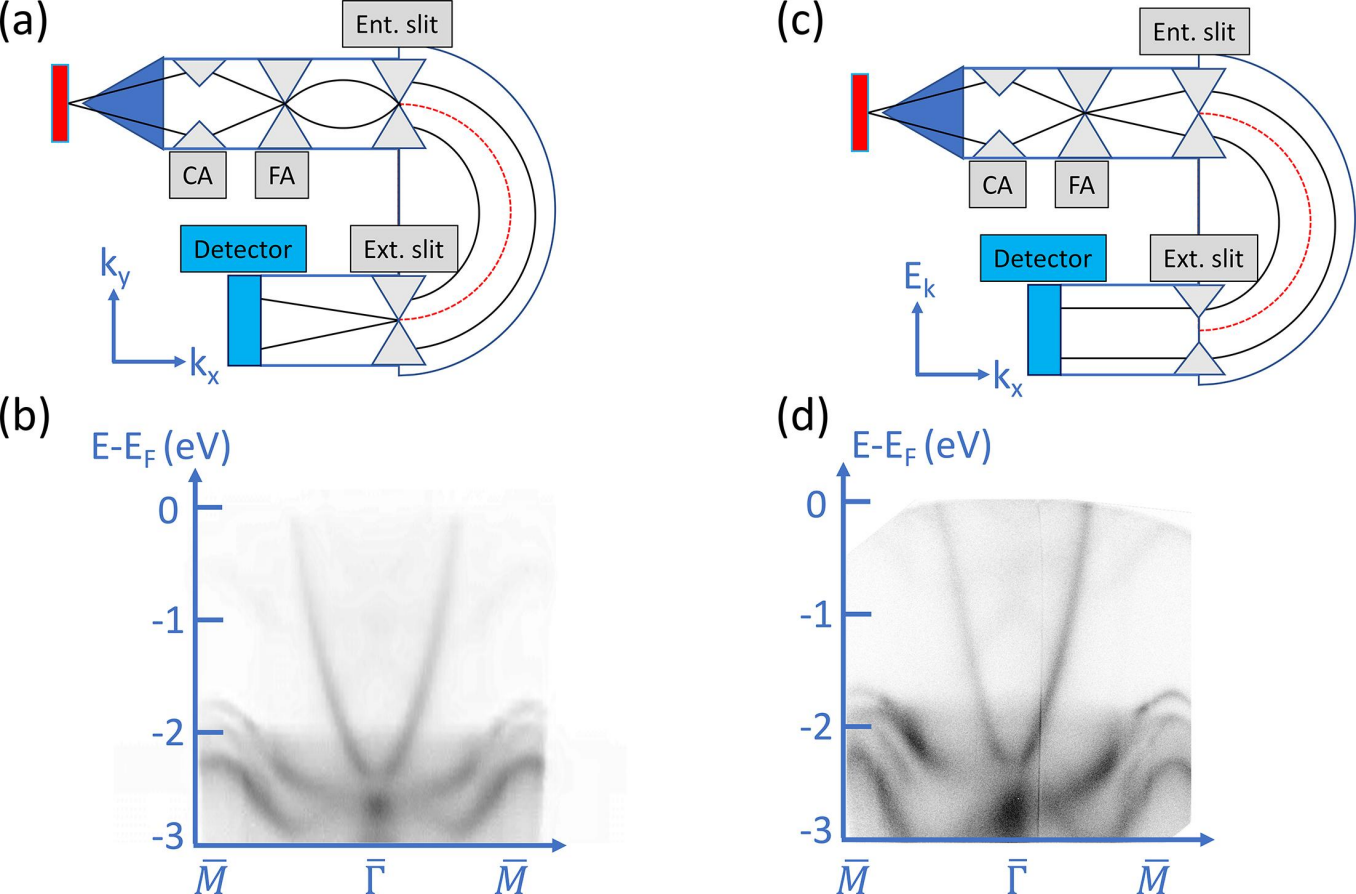
(*a*) The lens and aperture configuration of the constant energy contour mode. (*b*) An example of the Cu(001) band structure obtained from the 3D data set (*k*_*x*_, *k*_*y*_, *E*_*k*_) by the constant energy contour mode and sliced along the 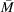 – 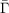 – 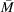 direction. (*c*) The lens and aperture configuration of ARPES mode. (*d*) An example of the Cu(001) band structure along the 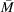 – 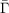 – 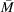 direction obtained during ARPES mode.

Our MM system is cable of switching between constant energy contour mode and ARPES mode. The constant energy contour mode projects the direct-space image onto the imaging plane at the FA to the entrance slit of the HEA as shown in Fig. 6 ▸(*a*). The momentum lens (MM lens) located after the exit slit of the HEA then transforms the energy-filtered direct-space image to a momentum-space image and projects it onto a two-dimensional (2D) detector. This gives rise to raw image data in the form of a plot of an electronic momentum (*k*_*x*_, *k*_*y*_) image at a selected kinetic energy. To obtain the energy dispersion relation or electronic band structure along a specific high-symmetry direction, a series of momentum images at different kinetic energies can be measured and stacked together to construct a 3D data set, as shown previously in Fig. 3 ▸, and then sliced along a target symmetry direction. Fig. 6 ▸(*b*) shows an example of slicing along the 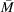 – 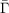 – 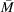 direction measured on a Cu(001) single crystal, where the total measuring time for the entire 3D data set, averaged from 100 repeated scans over a 3 eV range with increments of 20 meV, is about four hours.

In contrast, the ARPES mode of the momentum microscope projects the momentum image at the position of the contrast aperture (CA) in relatively large size to the entrance slit of the HEA, as shown in Fig. 6 ▸(*c*). After passing through the HEA, the photoelectrons at selected *k*_*y*_ have energy dispersion along each *k*_*x*_ at the opened exit slit, where the (*E*_*k*_, *k*_*x*_) at selected high-symmetry axis is obtained. The momentum lens after the exit slit only projects the (*E*_*k*_, *k*_*x*_) image to the detector as shown in Fig. 6 ▸(*d*) as an example along the same direction in Fig. 6 ▸(*b*). The constant energy contour mode utilizes a round entrance slit with a typical size of 0.2 mm to 2.0 mm in diameter and a comparably sized exit slit. In contrast, the ARPES mode utilizes a rectangular entrance slit of size 0.2 mm × 25 mm and a fully opened exit slit. The measuring time for a single spectrum, averaged from 600 repeated scans shown in Fig. 6 ▸(*d*), was about 10 min. However, although the constant energy contour mode has an increased acquisition time, this allows for the full 2D momentum parallel pattern to be recorded. One should note that the MM operates in *k*-space, recording energy versus parallel momentum patterns. In contrast, the conventional ARPES operates in real-space recording energy versus θ patterns. It should also be noted that the spectrum obtained directly from the ARPES mode [Fig. 6 ▸(*d*)] is slightly distorted compared with the one obtained from the contour mode. This is likely due to the fact that the focusing lens of the spin rotator located after the exit slit not being optimized when a fully opened exit slit is used during the ARPES mode.

## 6. Summary and perspectives

A photoelectron MM is now hosted at the TPS 27A2 endstation. Offline commissioning has demonstrated its capabilities for direct-space imaging via work function difference and momentum-space imaging with constant energy contour, respectively, using an Hg lamp or He(I) radiation. By recording momentum contour images at various kinetic energies, the electronic structure along all symmetry directions can be obtained simultaneously. ARPES mode is also available on the same instrument, providing an efficient way to measure the energy dispersion relation along a specific symmetry direction, if the sample surface symmetry is known in advance. In future, we would like to explore the capability of the microscope using a soft X-ray regime covered by the TPS 27A Soft X-ray Nanoscopy beamline. The photon source of the TPS 27A is powered by an elliptically polarized undulator and an active-mirror plane-grating monochromator system, which can deliver a photon energy between 90 eV and 3000 eV with tunable polarization, and a resolving power above 10000 over a wide spectral range. In addition to the capabilities mentioned above, the imaging based on various contrasts, such as X-ray photoelectron spectroscopy, X-ray absorption spectroscopy and X-ray magnetic circular dichroism will also be accessible in the same instrument, providing comprehensive information on element-resolved and spin-resolved features, when the synchrotron X-ray is available in the near future.
